# Printable Hydrogels Based on Alginate and Halloysite Nanotubes

**DOI:** 10.3390/ijms23063294

**Published:** 2022-03-18

**Authors:** Giuseppe Cavallaro, Lorenzo Lisuzzo, Giuseppe Lazzara, Stefana Milioto

**Affiliations:** Dipartimento di Fisica e Chimica, Università degli Studi di Palermo, Viale delle Scienze, pad. 17, 90128 Palermo, Italy; giuseppe.cavallaro@unipa.it (G.C.); giuseppe.lazzara@unipa.it (G.L.); stefana.milioto@unipa.it (S.M.)

**Keywords:** halloysite, alginate, hydrogel, beads, drug delivery, morphology

## Abstract

The design of hydrogels for the controlled release of active species is an attractive challenge. In this work, we prepared hybrid hydrogels composed of halloysite nanotubes as the inorganic component, and alginate as the organic counterpart. The reported procedure allowed us to provide the resulting materials with a peculiar wire-like shape. Both optical and scanning electron microscopy were used to characterize the morphological properties of the hydrogel wires, whose diameters were ca. 0.19 and 0.47 mm, respectively. The possibility to be exploited as drug delivery systems was carried out by loading the nanoclay with salicylic acid and by studying the release profiles. Thermogravimetric experiments showed that the amount of encapsulated drug was 4.35 wt%, and the salicylic acid was thermally stabilized after the loading into the nanotubes, as observed by the shift of the degradation peak in the differential thermograms from 193 to 267 °C. The kinetics investigation was conducted using UV–Vis spectrophotometry, and it exhibited the profound effects of both the morphology and dimensions on the release of the drugs. In particular, the release of 50% of the payload occurred in 6 and 10 h for the filiform hydrogels, and it was slower compared to the bare drug-loaded halloysite, which occurred in 2 h. Finally, an induction period of 2 h was observed in the release profile from the thicker sample.

## 1. Introduction

In recent times, research on hydrogels has been attracting the interest of the scientific community, as revealed by the growing number of published articles, which reached ca. 2700 units in 2016 [1]. Basically, hydrogels are defined as a unique class of three-dimensional cross-linked polymeric networks which possess the ability to retain a large amount of either aqueous solvent or biological fluids within their structures [2]. Their swelling ability, pH sensitivity, and temperature and/or other stimuli responsiveness represent some special properties of this class of materials which can be exploited in a wide range of technological applications [3,4]. For instance, the literature reports many studies where hydrogels can be applied in agriculture, pharmaceuticals, drug delivery systems, tissue engineering, food additives, diagnostics, biosensors, wound dressings, etc., [5,6,7,8,9,10].

Many materials, both naturally occurring and synthetic, can be used for the design of hydrogels, which are generally composed of different building blocks such as monomeric backbones of the (bio-)polymeric chain, cross-linking species, comonomers and a solvent to start swelling and network formation [11]. Depending on their properties and components, hydrogels can be classified in different ways. Based on composition, it is possible to define homopolymeric, copolymeric and multipolymeric hydrogels [2,12]. According to their physical structure, they can be categorized as amorphous, semicrystalline and crystalline materials. In addition, they can be classified as non-ionic (neutral), ionic (anionic or cationic), amphoteric or zwitterionic networks based on the charge on the crosslinked chains [13]. The nature of the crosslink junctions allows for the further separation of chemically cross-linked with permanent covalent bonds from physically cross-linked hydrogels, where ionic interactions, hydrogen bonds, or hydrophobic interactions play a major role [14]. Finally, hydrogels can also be defined according to their aspects as films, beads, matrix, etc., [2].

Among the various counterparts used in the preparation strategy, nanoparticles such as nanoclays hold a certain importance [15,16]. Halloysite nanotubes (HNTs) are naturally occurring aluminosilicates possessing a hollow tubular shape in the nanometric scale and opposite charges between the external surface, which is negative, and the internal surface, which is positive [17,18,19,20]. The difference in charge is a direct consequence of the chemical composition [21,22]. Halloysite is composed of a layer of a Si-centred tetrahedra overlapped to a layer of an Al-centred octahedra in a kaolinite-like structure [23]. Then, this kaolinite sheet rolls up to confer the nanoclay and its peculiar morphology and charge separation, bearing Si-O-Si groups outside and Al-OH groups in the inner lumen [23]. The dimensions of the nanotubes depend on the natural deposit, but generally, the length is in the 0.5–2 µm range, the external diameter is 50–200 nm and the internal diameter is 15–50 nm, respectively [24].

Due to their properties, HNTs can be used in many fields [25,26,27,28,29,30]. The possibility to encapsulate active molecules within their lumen has paved the way for applications in catalysis, food packaging, biomedical technology and cultural heritage treatment [31,32,33,34,35,36,37].

In this work, we designed a novel class of printable bio-hybrid hydrogels composed of alginate, as an organic counterpart and polymeric backbone, and halloysite nanotubes as inorganic fillers and nanocontainers for the loading and release of drugs. 

Alginate and HNT-based hydrogels have already been reported in the literature, as they represent a class of materials which can be used in the most diverse applications. For instance, they have been exploited for the removal of dyes and metals from water in the environmental field, for the delivery of drugs and anticancer compounds in biomedical technology, for the release of fertilizers in agriculture, and as scaffolds in tissue engineering [38,39,40,41,42].

Here, we tried to move a step forward by providing the designed systems with a novel and particular shape which differs from the usually prepared spherical beads. The proposed preparation protocol allowed us to reach the formation of filiform hydrogels. The physical aspect is one of the most important features since the applicability of hydrogels also depends on their shape, and a peculiar morphology can widen the range of possible uses. This factor is at the basis for the rapid development of three-dimensional printing of hydrogels, which represents an advanced manufacturing technology for fabricating 3D objects with complex structures to mimic native organs, functional tissues, etc., [43]. Unfortunately, 3D-printed objects produced from pure hydrogels possess both low mechanical strength and viscosity, which can be improved by the addition of inorganic nanofillers [44]. 

In this study, hydrogels with a wire-like three-dimensional structure and improved printability were developed for the controlled release of active species, and salicylic acid was employed as a model drug. Besides hydrogel wires and hydrogel beads were prepared for comparison. 

## 2. Results

### 2.1. HNTs Loaded with Salicylic Acid

**Figure 1:**
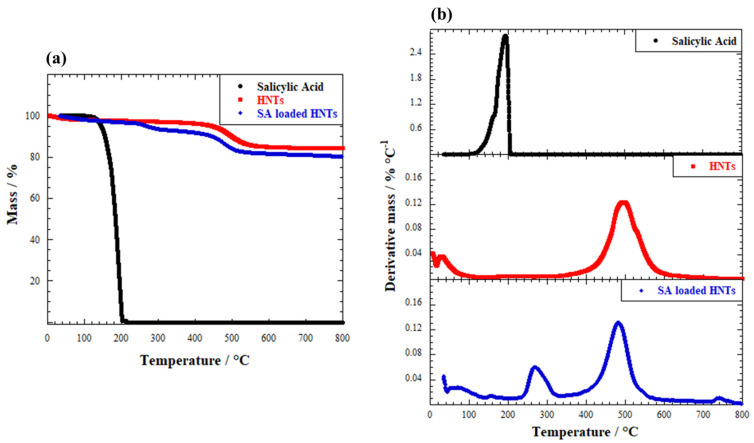
(**a**) Thermogravimetric (TGA) and (**b**) differential thermogravimetric (DTG) curves of pure salicylic acid, pristine HNTs and SA-loaded HNTs.

The first step for the design of printed hydrogels in drug delivery was the encapsulation of an active compound, namely salicylic acid, within the inner cavity of halloysite nanotubes by a vacuum-optimized procedure. The loading efficiency was investigated by thermogravimetric analysis (TGA). In particular, Figure 1a compares the thermograms of pure salicylic acid (SA), pristine halloysite nanotubes (HNTs) and SA-loaded HNTs (SA-HNTs). 

It can be observed that salicylic acid completely degrades in the range of temperature between 150–200 °C, where it undergoes first a decarboxylation to produce phenol, and then it decomposes, and its residue is 0% at higher temperatures [45,46]. This behaviour is typical in organic species, where intramolecular bonds are rapidly broken, and molecules are easily converted to CO_2_. Contrarily, halloysite nanotubes display a much higher residue, even at 800 °C, due to their inorganic nature. Only one thermal degradation step can be observed for halloysite in the 400–500 °C range, and it is related to the loss of structural hydroxyl groups present on the surface of the clay mineral, as described in previous studies [47]. Finally, the thermogram of salicylic acid-loaded halloysite shows a mass loss higher than pure SA but lower than HNTs at 800 °C. Being composed of both organic and inorganic moities, the former ones are easily degraded, thus leaving a skeleton of inorganic matter. This is the first proof of the encapsulation of salicylic acid within the clay. In order to quantitatively evaluate the loading efficiency, the rule of mixtures was applied to pure SA, neat HNTs and SA-HNTs composite thermal data by focusing the mass loss at 150 °C (ML150) and the mass residue at 800 °C (MR800) [48]. See Table 1. 

The mass loss at 150 °C can be attributed to the evaporation of adsorbed water, and it is a direct measurement of the samples’ proper moisture content. As previously discussed, the mass residue at 800 °C is the final mass of the samples after reaching the highest temperature. Salicylic acid is completely degraded, HNTs residue is ca. 84% due to the inorganic nature, and SA-loaded HNTs residue is 80.4 wt% due to the decomposition of SA moiety. According to the rule of mixtures, the loading efficiency was calculated by thermal data, and the results were 4.35 wt%, which is in good agreement with the literature for vacuum-optimized protocols. A similar efficiency was reported for salicylic acid-loaded HNTs under the same experimental conditions (P = 0.01 atm) [48]. Moreover, the analysis of differential thermograms (DTG) provided some interesting insights about the thermal stabilization. DTG curves are reported in Figure 1b. Although the dehydroxylation of halloysite occurs at the same temperature in pristine clay and SA-loaded HNTs, namely ca. 485 °C as calculated by using the value of the peak maximum, some variations can be observed for salicylic acid. The DTG curve of pure SA displays a peak at 193 °C, whereas this peak is shifted to 267 °C in the SA-loaded HNTs sample. This effect is most likely related to the thermal stabilization of the drug after its encapsulation within the inner lumen of the clay. The literature reports that the loading of organic species into the inner space of HNTs is responsible for their thermal stabilization, since they are more protected in the confined space compared to the free molecules [49].

### 2.2. Preparation of ALG/HNTs Hydrogel Wires

The preparation of alginate/halloysite hydrogel wires was carried out by using a peristaltic pump to flow the dispersion through a solution of calcium chloride, as described in the experimental section (Section 4.2.2). To reach the particular shape, hydrogels were formed inside tubes with different diameters (0.38 and 0.76 mm) which acted as templates. 

**Figure 2:**
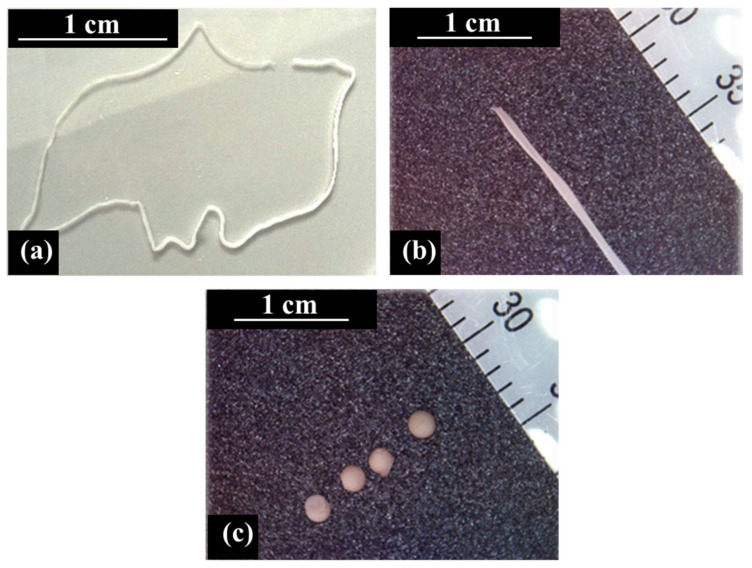
Optical images of (**a**,**b**) hydrogel wires and (**c**) hydrogel beads.

Figure 2 shows the optical images of the prepared samples, whereas their dimensions are reported in Table 2 together with the diameter of the tube employed in the preparation procedure. Hydrogel beads were also prepared for comparison by the dropping technique. 

The proposed preparation protocol allowed us to create printable hydrogels with a tailored shape whose dimensions can be tuned by varying the experimental set up. In particular, the size of the hydrogel wires increases from 0.19 to 0.47 mm in diameter when the size of the tube increases from 0.38 to 0.76 mm, respectively.

As these samples are completely formed within the inner space of the peristaltic pump tube, where Ca^2+^ ions enter and crosslink the alginate chains, the dimensions of the wires are smaller than those of the external template. Contrarily, the diameter of the hydrogel beads is larger than the tube size, being 1.26 mm compared to 0.38 mm, and their final shape is spherical, as reported in previous studies [50].

**Figure 3:**
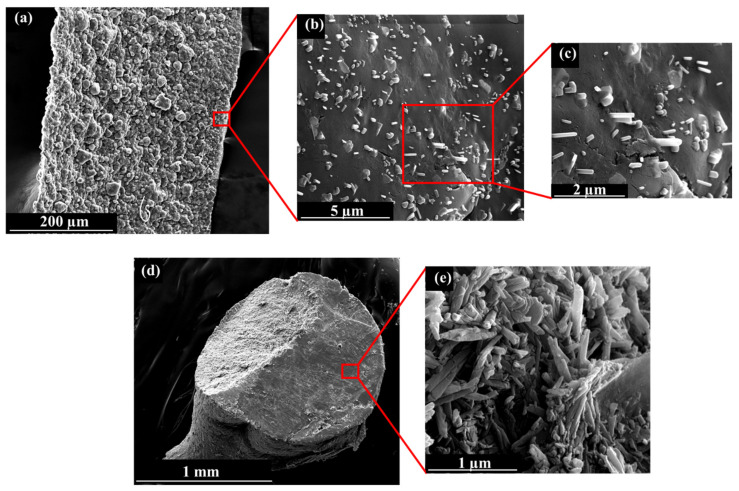
Micrographs of (**a**–**c**) hydrogel wires and (**d**,**e**) hydrogel beads at different magnifications.

Aimed at investigating the morphology of the hydrogel wires, scanning electron microscopy was performed. Micrographs are reported in Figure 3.

The filifom shape of the prepared hydrogels can be observed by SEM images (Figure 3a), with an overall width of 370 µm, which is in good agreement with the statistical analysis carried out on optical images, as reported in Table 2 for the sample Hydrogel wires_2. The images at higher magnifications show the peculiar distribution of halloysite, which is partially inserted inside the biopolymeric matrix, with a certain portion of each nanotube emerging and protruding on the external surface (Figure 3b,c). Besides, the micrographs also allow us to confirm the typical dimensions of HNTs, which are about 1–2 µm in length and whose external diameter is 100–200 nm, as reported in the literature [51].

For comparison, the SEM images of hydrogel beads are also reported where their spherical shape can be observed (Figure 3d). Herein, contrarily to the hydrogel wires, the clay nanotubes are homogeneously distributed and randomly dispersed, without any orientational effect (Figure 3e).

### 2.3. Release Studies

**Figure 4:**
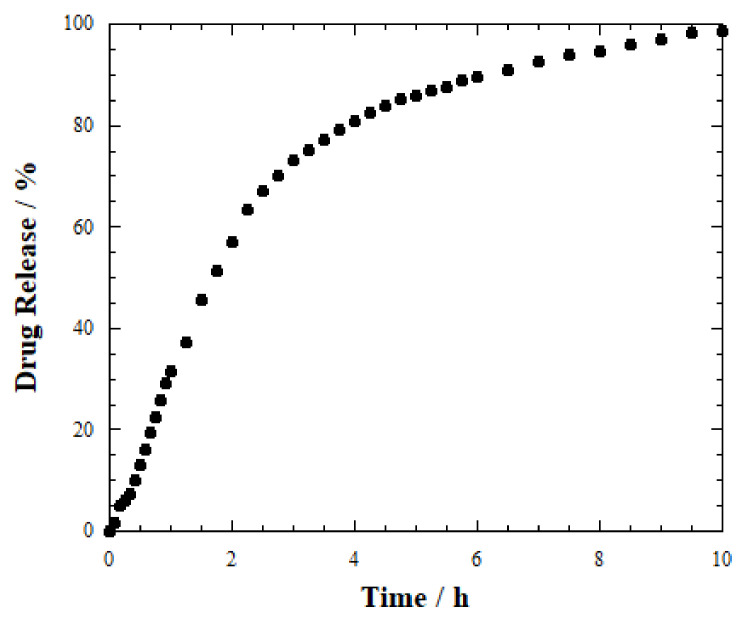
Release profile for SA-loaded HNTs tablet.

Kinetics investigation was conducted by UV–Vis spectrophotometry to exhibit any possible effect of the morphology on the drug release from the prepared materials. First, a tablet made of salicylic acid-loaded HNTs was focused (Figure 4). 

It can be observed that the release profile displays a burst effect in the initial steps, and then the trend becomes more constant. In particular, 50% of salicylic acid is released in the aqueous medium after ca. 2 h. Drug delivery is quantitative after 6 h, being 90% of the payload released. Then, 100% is reached after 10 h. In light of this, it is possible to assert that the kinetics is fast and that SA is easily delivered by the nanotubes, being fully available in a short time. Compared to the dissolution of pure salicylic acid, which was completely detected in the solution already at the first experimental point, the release profile of SA-loaded HNTs is further proof for the drug encapsulation within the nanoclay. It should be noted that the incorporation into the lumen of halloysite generates a sustained release profile due to confinement and tortuosity effects [52].

**Figure 5:**
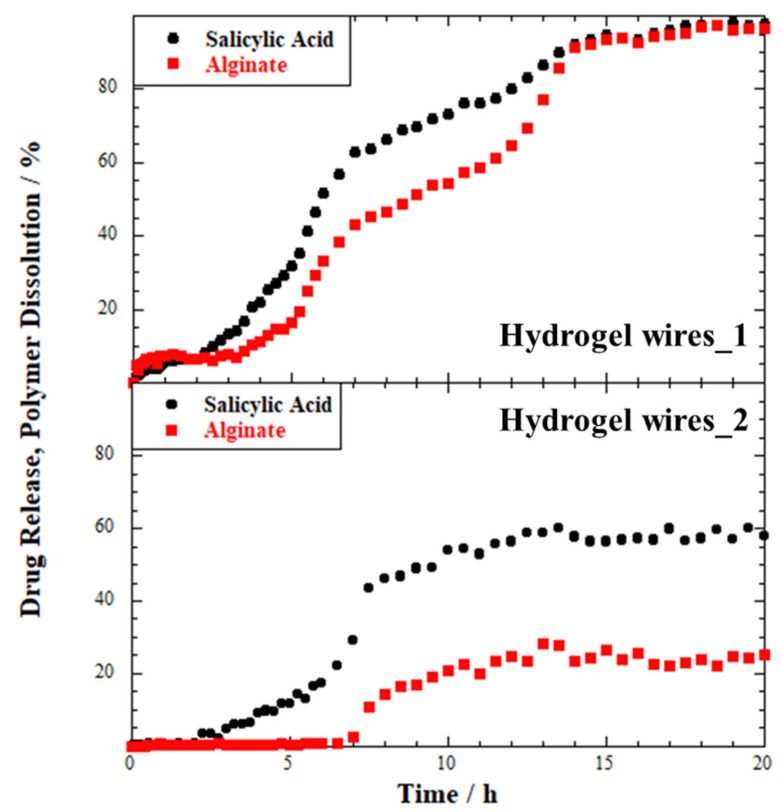
Release profile for SA-loaded Hydrogel wires_1 and Hydrogel wires_2 samples.

Thereafter, the release profiles were recorded for the hydrogel wires samples (Figure 5). In these cases, both the drug release and the polymer dissolution were considered.

Concerning Hydrogel wires_1, both the SA release and the alginate dissolution start once the sample is in contact with the medium. More specifically, 6% and 7% of the drug and the biopolymer can be detected in the environment after 1 h, respectively. Salicylic acid release is 50% after 6 h, and the maximum value, namely the release of 97.5% drug, is reached in 18 h. The biopolymer dissolution reached a similar extent in the same range of time. Conversely, an induction period can be observed in the kinetics profiles of Hydrogel wires_2, which is thicker than the previous sample. No drug can be released within 2 h from contact with the aqueous medium, and no biopolymer dissolution occurs within 6 h. The presence of this induction deeply affects the kinetics of the process, and 50% of the drug is delivered only after ca. 10 h. Contrary to the hydrogel wires with smaller diameter, in this case, the SA release reaches a maximum value of 59% after 13 h, and then a constant trend is maintained even at longer times. The alginate dissolution displays a similar profile, reaching it highest value of 28% in 13 h followed by a constant value as a function of time. These effects can be most likely related to the morphological features of the samples, to the presence of the alginate, and to the different diameters, as discussed in the next paragraph.

**Figure 6:**
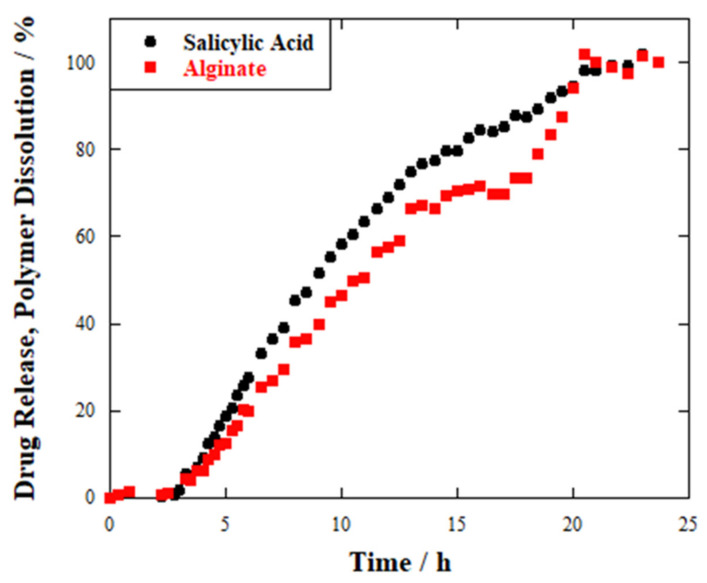
Release profile for SA-loaded hydrogel beads.

For comparison, the release profile of salicylic acid was also recorded from spherical beads (Figure 6).

The overall behaviour of the hydrogel beads sample can be described as the average of the Hydrogel wires_1 and Hydrogel wires_2 samples. For instance, an induction time of 3 h can be observed in the initial part of its profile, since neither drug release nor polymer dissolution can be detected. Then, an increasing amount of salicylic acid can be delivered over time, reaching the value of 50% after 9 h. Finally, the quantitative detection of the drug in the environment occurs after 20 h, being the amount of released payload ca. 98%. Alginate dissolution displayed a similar profile. 

Aimed at evaluating the different kinetic behaviours, the kinetics parameters are reported in Table 3 to enlighten both similarities and differences in the drug delivery from each sample. T_50%_ is the time needed for the delivery of 50% of the payload to the release medium.

It is clear that the morphology plays a crucial role in the release kinetics, which is slowed down in the hydrogel samples (i.e., both filiform wires and spherical beads) compared to SA-loaded HNTs tablets. In addition, the thickness of the wires allows us to control and to tune the process rate. 

## 3. Discussion

The proposed procedure allowed us to reach a loading efficiency of 4.35 wt% for salicylic acid encapsulated within the lumen of halloysite nanotubes. The confinement of salicylic acid within the inner volume of the nanoclay conferred higher thermal stability to the drug, as pointed out by DTG analysis. The degradation step is shifted towards a higher temperature, namely from 193 to 267 °C. This effect has already been reported in previous studies, and it can be explained by considering the hollow nanotubular shape of halloysite which can act as a barrier towards volatile products, gases and heat transfer, and as a protective shell for the thermal decomposition of the organic matter [49].

The preparation strategy enabled the formation of hydrogels with peculiar features, and three-dimensional filiform systems were created. In particular, the dimensions of the hydrogel wires can be tailored by changing the diameter of the peristaltic pump tube. As a result, wires with different diameters were prepared, and namely Hydrogel wire_1 with 0.19 mm and Hydrogel wire_2 with 0.47 mm in diameters, respectively. 

To do so, SA-loaded HNTs/alginate dispersions flow through the tube, and they meet the calcium chloride solution. Hence, the Ca^2+^ presence is responsible for activating the crosslinking of the polymeric chains, thus creating the hydrogels from a restricted and confined space, and the peristaltic tube works as a moulder.

During this process, HNTs keep their homogeneous distribution in the biopolymeric matrix, and the final result is the partial entrapment of nanotubes in the network, with some parts of each tube immersed inside the hydrogel and some parts protruding on the external surface, as observed by scanning electron microscopy. The preparation protocol did not alter the hollow nanotubular shape of the clay. In the case of hydrogel beads, the nanoclay is randomly dispersed without protruding from the sample; instead, they are well accommodated on the external surface.

The peculiar morphology of the hydrogel wires plays a major role in the release kinetics of salicylic acid, which is still encapsulated inside the inner lumen of the nanoclay. Regardless of their dimensions, both the Hydrogel wires_1 and Hydrogel wires_2 samples showed a more sustained and controlled release of drugs compared to the SA-loaded HNTs. For instance, the time needed to release 50% of the salicylic acid payload is 6 h for Hydrogel wires_1, 10 h for Hydrogel wires_2 and only 2 h for the SA-HNTs tablet. This effect is related to the presence of the alginate which forms the gel upon swelling with water in the presence of Ca^2+^ ions, thus slowing down the rate of the process and the drug exit from the HNT inner lumen. Similar findings were observed for the hydrogel beads, which release 50% of SA within 9 h.

An effect of the dimensions was also exhibited. The Hydrogel wires_2 displayed a slower release of salicylic acid over time compared to its thinner counterparts, namely the Hydrogel wires_1. This effect is most likely related to the width and structural organization of the polymeric matrix and to the distribution of halloysite which is partially inserted within the filiform samples. Once released from HNTs, salicylic acid can become further entrapped within the gel network, by interacting with the alginate, for a certain range of time before being available in the external aqueous medium. This delayed effect is related to the specific diameter of the filiform sample, becoming larger as the size increases. An induction time which is dependent on the wires dimensions was also registered in the profiles. Namely, 6% of salicylic acid is delivered by the Hydrogel wire_1 sample after 1 h, whereas no drug can be released within a 2 h induction period by the Hydrogel wire_2 sample. Similar observations were made for the doxycycline release from core/shell gel beads made of chitosan and alginate but also for the release of active compounds from nanocomposite films and tablets as structured materials [50,51,53].

## 4. Materials and Methods

### 4.1. Materials

Halloysite nanotubes, HNTs (Al_2_Si_2_O_5_(OH)_4_·2H_2_O) were a gift from Imerys Ceramics. Sodium alginate (ALG, Mw = 70–100 kg mol^−1^) is a Sigma Aldrich product. Salicylic acid (SA) and calcium chloride dihydrate (CaCl_2_·2H_2_O, ≥99%) are from Fluka and Panreac, respectively. All the products were used without any purification treatment.

### 4.2. Methods 

#### 4.2.1. Loading of Salicylic Acid into HNTs

In order to encapsulate SA within the lumen of halloysite nanotubes, a saturated solution of drug in water was prepared, and the clay was added as a powder in a HNTs/SA ratio of 2:1. Then, the dispersion was magnetically stirred for 24 h at room temperature. According to our previous work, the system was subjected to three vacuum cycles of 30 min each, in a vacuum jar. This step was introduced to optimize the drug loading inside the clay [48]. Between each step, the dispersion was vortexed for 5 min for better homogenization. Finally, the drug-loaded HNTs were separated by using a centrifuge (10 min at 8000 rpm), washed with water three times to remove any excess and dried overnight at 40 °C. Prior to any use, the samples were smashed to powder in a mortar.

#### 4.2.2. Preparation of ALG/HNTs Hydrogel Wires

**Figure 7:**
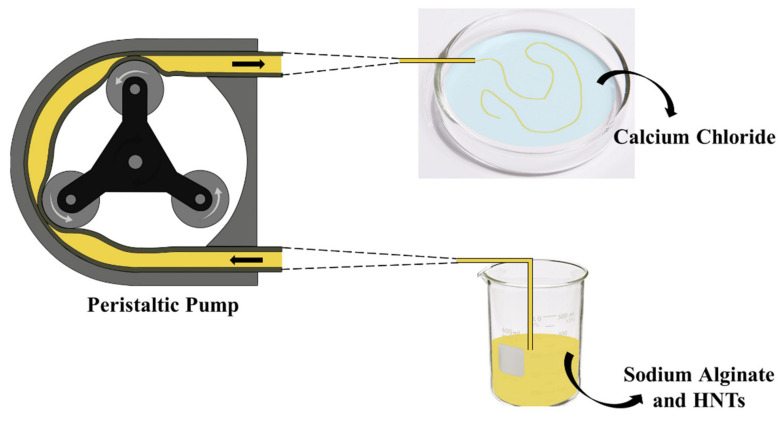
Schematic representation of the overall procedure.

The preparation of the hydrogel wires was carried out by using a peristaltic pump (Gilson Minipuls 3) to flow the ALG/HNTs dispersion and to provide the proper morphology. In particular, an aqueous solution of sodium alginate (2 wt%) was prepared, and halloysite was loaded with salicylic acid and was added at a biopolymer/nanoclay ratio of 1:1. The dispersions were sonicated for 10 min and kept under stirring overnight at room temperature to reach high stability. Then, by means of the peristaltic pump (rate: 999 rpm, flow: 0.0027 g s^−1^), each dispersion flowed through a tube with a certain diameter to a Petri dish containing a CaCl_2_ 0.1 M solution. Once the ALG/HNTs dispersion exits the tube, Ca^2+^ ions act as cross-linking species to form the gels in the aqueous media. Finally, the samples were washed and dried. Two pump tubes with different diameter (0.38 mm and 0.76 mm) were used to change the thickness of the hydrogel wires. Figure 7 reports a schematic representation of the overall procedure.

#### 4.2.3. Preparation of ALG/HNTs Hydrogel Beads

The ALG/HNTs hydrogel beads were prepared by using the dropping technique [50]. In this case, the peristaltic pump was used to drop the biopolymer/nanoclay dispersions (sodium alginate concentration of 2 wt% and ALG/HNTs ratio 1:1) into the aqueous solution of CaCl_2_ 0.1 M placed in a Petri dish to cross-link the alginate. The distance from the needle to the liquid surface was 2 cm, and the tube diameter was 0.038 mm. After 10 min of contact, the resulting hydrogel beads were taken and dried out at 40 °C overnight.

#### 4.2.4. Release Kinetics of Salicylic Acid

The study of release kinetics was conducted by recording profiles in water using UV–Vis spectroscopy. In particular, 0.01 g of each sample (hydrogel wires or hydrogel beads) was weighted and directly placed into quartz cuvettes. An amount of 2.5 cm^3^ of distilled water was added, and the spectra were recorded at pre-established time intervals for 24 h. In order to investigate both the release of salicylic acid and the dissolution of alginate, their two main bands were focused, being at 296 and 276 nm for SA and ALG, respectively. Calibration curves were recorded for both the drug and the biopolymer in quartz cuvettes under the same conditions. For release kinetics studies on salicylic acid-loaded halloysite, a tablet was made by subjecting 0.01 g of powder to 10 tons for 10 min. Concerning the dissolution of pure salicylic acid, the whole amount of pure drug could be detected in the solution already at the first experimental point.

#### 4.2.5. Characterization Techniques

Thermogravimetry (TG) measurements were performed by using a Q5000 IR apparatus (TA Instruments, Milan, Italy) under nitrogen flows of 25 and 10 cm^3^ min^−1^ for the sample and the balance, respectively. Each sample (approximately 5 mg) was heated from room temperature to 800 °C at a rate of 10 °C min^−1^. The loading efficiency was provided using the rule of mixtures on neat HNTs, pure SA and SA/HNTs composites [48]. The apparatus was calibrated by the Curie temperature of standards [54]. In order to study the release kinetics obtained from the prepared materials, UV–Vis spectra in quartz cuvettes were recorded by a Specord S600 (Analytik, Jena, Germany). Optical images were produced by using a DIGITUSs (DA-70351) microscope and processed by using the Digital Viewer software. Dimensions were measured by using ImageJ as software [55]. Scanning electron microscopy was conducted by using a microscope ESEM FEI QUANTA 200F. Before each experiment, each sample was coated with gold in argon by means of an Edwards Sputter Coater S150A to avoid charging under the electron beam. The measurements were carried out in high vacuum mode (<6 × 10^−4^ Pa) for the simultaneous secondary electron; the energy of the beam was 10 kV, and the working distance was 10 mm.

## 5. Conclusions

We prepared inorganic–organic hybrid hydrogels composed of halloysite nanotubes and alginate for drug delivery applications. The use of a peristaltic pump with tubes of different diameter in the experimental setup conferred peculiar features to the final materials. The proposed method allowed us to design printable hydrogels with novel three-dimensional wire-like shape with increasing and tuneable sizes. Namely, hydrogel wires with ca. 0.19 and 0.47 mm diameters were developed. The wire morphology and dimensions demonstrated to play a crucial role in the release of salicylic acid, which was previously loaded within the inner cavity of the nanoclay with a loading efficiency of 4.35 wt%. Besides being thermally stabilized by the encapsulation into the nanotubes, as observed by the shift of the peaks in the differential thermogravimetry measurements from 193 to 267 °C, the release kinetics of salicylic acid from the hydrogel wires is slowed down compared to the drug-loaded HNTs system without alginate. The release of 50% of the amount of encapsulated drug occurs in 2, 6 and 10 h for the HNTs-SA tablet, the Hydrogel wires_1 and the Hydrogel wires_2 samples, respectively.

Moreover, the different sizes of the hydrogel wires are responsible for a different induction period of 0 and 2 h for the Hydrogel wires_1 and the Hydrogel wires_2 materials. In this case, the release kinetics is slowed down for the ticker sample due do the structural arrangement of the components and to the interactions between the drug and the gel network. The effect of the morphology was also confirmed by the release kinetics studies conducted on hydrogel beads, which were prepared for comparison.

In conclusion, the proposed procedure allowed us to prepare hydrogel systems with a novel 3D complex structure, which is different from the usually reported spherical shape. These findings introduce new scenarios in the manufacturing of printable gels which could find application in the most diverse domains. As long as the reported filiform systems are concerned, their rheological behaviour needs to be investigated in order to evaluate some important properties, such as thixotropy and self-healing capability. In addition, the study of swelling behaviour of the hydrogel wires together with the assessment of their stability in media mimicking biologic environments and in vitro biocompatibility assays will be the main topic for further studies. 

## Figures and Tables

**Table 1:** Thermogravimetric parameters of salicylic acid, HNTs and SA-loaded HNTs.

Sample	ML150 (wt%)	MR800 (wt%)
Salicylic acid	6.80 ± 0.11	0
HNTs	2.17 ± 0.04	84.1 ± 1.2
SA-loaded HNTs	2.49 ± 0.04	80.4 ± 1.1

**Table 2:** Dimensions of tubes and dried samples diameters.

Sample	Tube Diameter (mm)	Average Diameter (mm)
Hydrogel beads	0.38	1.26 ± 0.06
Hydrogel wires_1	0.38	0.19 ± 0.03
Hydrogel wires_2	0.76	0.47 ± 0.15

**Table 3:** Kinetics parameters for the release of salicylic acid from the investigated materials.

Sample	T_50%_ (h)	Induction Period (h)
HNTs-SA tablet	2	0
Hydrogel wires_1	6	0
Hydrogel wires_2	10	2
Hydrogel beads	9	3

Error: ± 1%.
